# Supplementary material for: Classic Chromophobe Renal Cell Carcinoma Incur a Larger Number of Chromosomal Losses Than Seen in the Eosinophilic Subtype
Source: Cancers (Basel). 2019 Oct 3;11(10):1492. doi: 10.3390/cancers11101492 (PMC6826417; doi:10.3390/cancers11101492)
Supplement: Supplementary file 1 [file cancers-11-01492-s001.zip › cancers-580098-supplementary-final/cancers-580098-supplementary.docx]

**Supplementary Materials**

**Table S1.** Copy number variation (chromosomal losses) in classic and eosinophilic chromophobe renal cell carcinomas in TCGA-KICH cohort (Chr. = chromosome).

|  | **TCGA** | | | |
| --- | --- | --- | --- | --- |
| **Characteristics** | **n (%)** | **Classic chRCC ^a^ (%)** | **Eosinophilic chRCC ^b^ (%)** | ***p*-value** |
|  | 66 | 53 (80.3) | 13 (19.7) |  |
| Chr.1 status |  |  |  |  |
| Loss | 55 (83.3) | 48 (90.6) | 7 (53.8) | <0.01 |
| No loss | 11 (16.7) | 5 (9.4) | 6 (46.2) |  |
| Chr.2 status |  |  |  |  |
| Loss | 49 (74.2) | 44 (83.0) | 5 (38.5) | <0.01 |
| No loss | 17 (25.8) | 9 (17.0) | 8 (61.5) |  |
| Chr.6 status |  |  |  |  |
| Loss | 52 (78.8) | 47 (88.7) | 5 (38.5) | <0.001 |
| No loss | 14 (21.2) | 6 (11.3) | 8 (61.5) |  |
| Chr. 10 status |  |  |  |  |
| Loss | 49 (74.2) | 46 (86.8) | 3 (23.1) | <0.001 |
| No loss | 17 (25.8) | 7 (13.2) | 10 (76.9) |  |
| Chr.13 status |  |  |  |  |
| Loss | 45 (68.2) | 40 (75.5) | 5 (38.5) | 0.01 |
| No loss | 21 (31.8) | 13 (24.5) | 8 (61.5) |  |
| Chr.17 status |  |  |  |  |
| Loss | 50 (75.8) | 45 (84.9) | 5 (38.5) | 0.001 |
| No loss | 16 (24.2) | 8 (15.1) | 8 (61.5) |  |
| Chr.21 status |  |  |  |  |
| Loss | 35 (53.0) | 30 (56.6) | 5 (38.5) | n.s. |
| No loss | 31 (47.0) | 23 (43.4) | 8 (61.5) |  |
| Loss of any chromosome ^c^ |  |  |  |  |
| present | 54 (81.8) | 48 (90.6) | 6 (46.2) | <0.001 |
| absent | 12 (18.2) | 5 (9.4) | 7 (53.8) |  |

^a^ defined as presence of “pale cells”, ^b^ defined as absence of “pale cells”, ^c^ Loss of any: Loss of any chromosome among chr. 2, 6, 10, 13, 17 or 21; n.s.: not significant.
